# Skeletal muscle metabolic responses to physical activity are muscle type specific in a rat model of chronic kidney disease

**DOI:** 10.1038/s41598-021-89120-8

**Published:** 2021-05-07

**Authors:** Keith G. Avin, Meghan C. Hughes, Neal X. Chen, Shruthi Srinivasan, Kalisha D. O’Neill, Andrew P. Evan, Robert L. Bacallao, Michael L. Schulte, Ranjani N. Moorthi, Debora L. Gisch, Christopher G. R. Perry, Sharon M. Moe, Thomas M. O’Connell

**Affiliations:** 1 Division of Nephrology, Indiana University School of Medicine, 950 W. Walnut St., R2 202, Indianapolis, IN 46202 USA; 2 Department of Physical Therapy, Indiana University School of Health and Human Sciences, Indianapolis, IN USA; 3 Roudebush Veterans Affairs Medical Center, Indianapolis, IN USA; 4 School of Kinesiology and Health Science, Muscle Health Research Centre, York University, Toronto, ON Canada; 5 Department of Anatomy and Cell Biology, Indiana University School of Medicine, Indianapolis, IN USA; 6 Department of Radiology and Imaging Sciences, Indiana University School of Medicine, Indianapolis, IN USA; 7 Departamento de Engenharia Mecânica, Universidade Federal do Rio Grande do Sul, Porto Alegre, RS Brazil; 8 Department of Otolaryngology, Head and Neck Surgery, Indiana University School of Medicine, Indianapolis, IN USA

**Keywords:** Metabolomics, Chronic kidney disease, Mitochondria

## Abstract

Chronic kidney disease (CKD) leads to musculoskeletal impairments that are impacted by muscle metabolism. We tested the hypothesis that 10-weeks of voluntary wheel running can improve skeletal muscle mitochondria activity and function in a rat model of CKD. Groups included (n = 12–14/group): (1) normal littermates (NL); (2) CKD, and; (3) CKD-10 weeks of voluntary wheel running (CKD-W). At 35-weeks old the following assays were performed in the soleus and extensor digitorum longus (EDL): targeted metabolomics, mitochondrial respiration, and protein expression. Amino acid-related compounds were reduced in CKD muscle and not restored by physical activity. Mitochondrial respiration in the CKD soleus was increased compared to NL, but not impacted by physical activity. The EDL respiration was not different between NL and CKD, but increased in CKD-wheel rats compared to CKD and NL groups. Our results demonstrate that the soleus may be more susceptible to CKD-induced changes of mitochondrial complex content and respiration, while in the EDL, these alterations were in response the physiological load induced by mild physical activity. Future studies should focus on therapies to improve mitochondrial function in both types of muscle to determine if such treatments can improve the ability to adapt to physical activity in CKD.

## Introduction

Chronic kidney disease (CKD) is a worldwide epidemic affecting over 30 million Americans, or 1 in 10 individuals^1^. CKD progression can lead to significant adverse events including falls and fractures that are associated with impaired musculoskeletal health^2–4^. Impaired musculoskeletal health has been shown to progress as CKD progresses by impacting skeletal muscle size, performance and exercise capacity^5–10^. Physical activity and exercise performance, in particular, are thought to decline in CKD due to sarcopenia that has been attributed to cellular dysfunction including increased muscle catabolism linked to increased oxidative stress, altered renin-angiotensin signaling and mitochondrial dysfunction^11–13^.

Physical activity is a term that encompasses all bodily movements (i.e., walking, cycling, active recreation), performed for enjoyment, regardless of skill level^14^. Exercise falls under the umbrella of physical activity and is planned and structured repetitive movements intended to improve health. These components are the basis of exercise prescription that targets objective improvement or maintenance in physical health and/or fitness. Physical activity, as an umbrella term, is commonly recommended for a number of diseases that cause musculoskeletal or metabolic dysfunction, such as sarcopenia with aging^15,16^, diabetes^17,18^, and cardiovascular disease^18,19^. Physical inactivity is associated with increased mortality and hospitalizations, and thus increased activity is encouraged for all^20–22^. However, studies of exercise in CKD have yielded variable results in clinical^23–26^, and preclinical CKD models^27–30^. In our animal model of CKD, we demonstrated that forced treadmill running induced oxidative stress and muscle catabolism^28^, whereas modest improvements occurred with voluntary wheel running^7^. In contrast, voluntary wheel running, a parallel form of exercise to walking in humans, was more effective in improving musculoskeletal health^7^. These differences suggest that exercise and physical activity, may have differential effects on muscle metabolism, but further exploration is needed to identify the underlying mechanisms.

In the present study, we sought to investigate the role that CKD and physical activity have upon muscle metabolism by directly assessing mitochondrial function using metabolomics and tissue respirometry. We previously have studied the effects of voluntary wheel running upon serum biochemistry (i.e., creatinine, phosphorous, and parathyroid hormone), physical function and musculoskeletal adaptation^7^. We expanded the current analyses to test the hypothesis that 10-weeks of voluntary wheel running improves skeletal muscle mitochondrial function in a rat model of CKD. We are using the term physical activity because wheel running was performed in a voluntary manner void of goal-oriented dosing. The metabolomics analyses include a large targeted, mass-spectrometry based analysis of the primarily slow-twitch soleus and mostly fast-twitch EDL tissues. The targeted panel enabled a quantitative analysis of approximately 180 metabolites from a wide range of chemical classes including amino acids, acylcarnitines, biogenic amines, and an extensive coverage of lipids. In each of the skeletal muscle tissue types, we evaluated the functional properties of mitochondria by measuring mitochondrial respiration rates. This technique has been utilized in age-related and CKD-related mitochondrial dysfunction^31–33^. We utilized these techniques to assess metabolic changes associated with CKD and physical activity in normal littermates, CKD rats and CKD-rats with voluntary wheel running. The inclusion of both primarily slow- and fast-tissue types is important given fast muscle types are more susceptible to atrophy and loss of force with general aging. As there is conflicting evidence regarding muscle type alterations in CKD^10,34^ a greater understanding of the metabolic influences of each muscle type can inform and guide appropriate therapies.

## Results

### Metabolic signatures of CKD and CKD-W rat soleus

**Figure 1:**
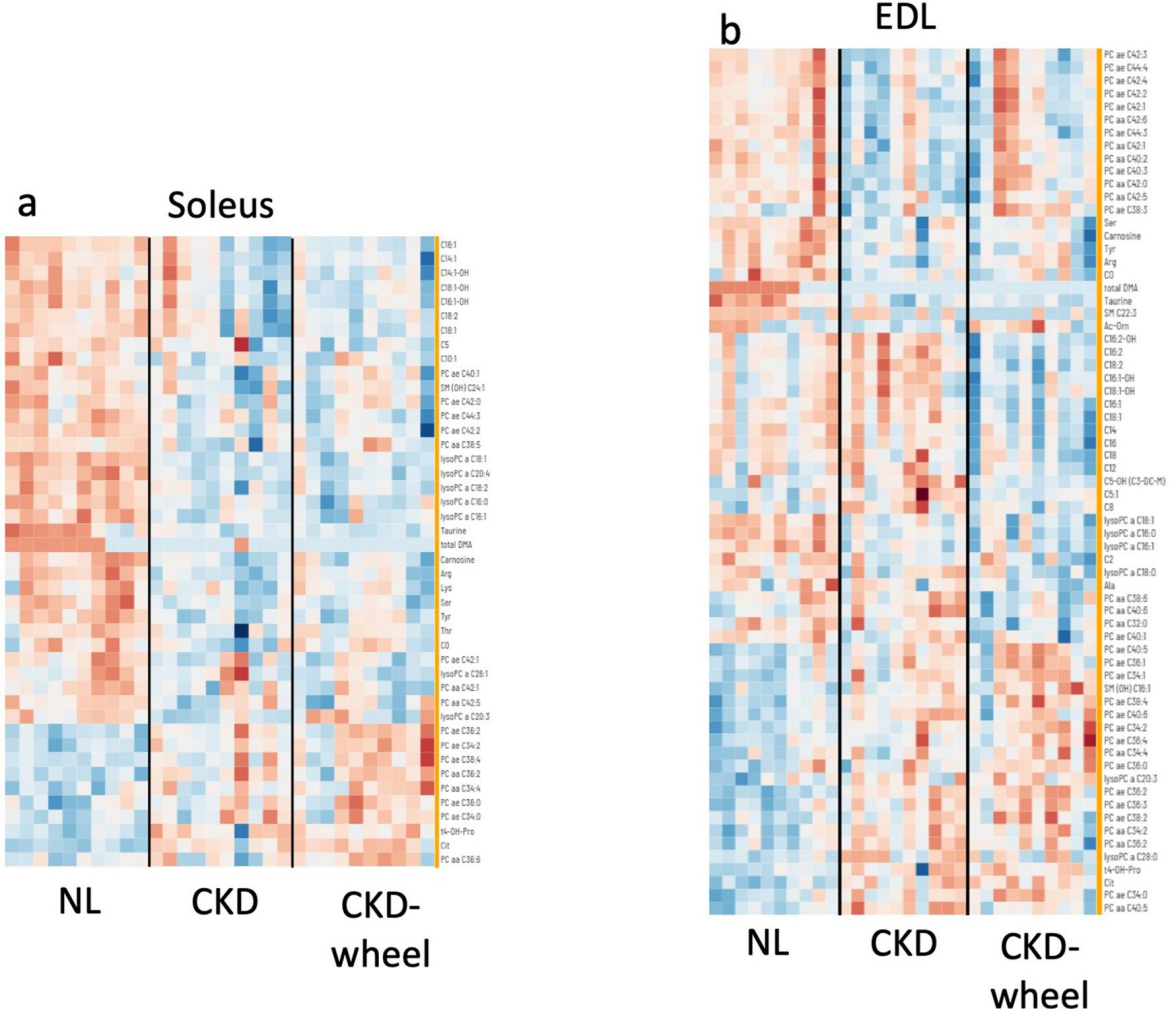
Heatmaps of metabolomics data from the soleus and EDL. The heatmaps include the significantly altered metabolites in each tissue presented as z-scores for (**a**) soleus and (**b**) EDL. The metabolites are ordered with K-nearest neighbors hierarchical clustering.

Extracts of the soleus were analyzed by MS-based metabolomics and Fig. 1 shows the heatmap of significant metabolic alterations found in the soleus (left panel) and EDL (right panel). The results revealed 44 metabolites that were significantly altered in one of the groups with 33 differences between NL and CKD, 35 between NL and CKD-W and no differences between CKD and CKD-W.

**Figure 2:**
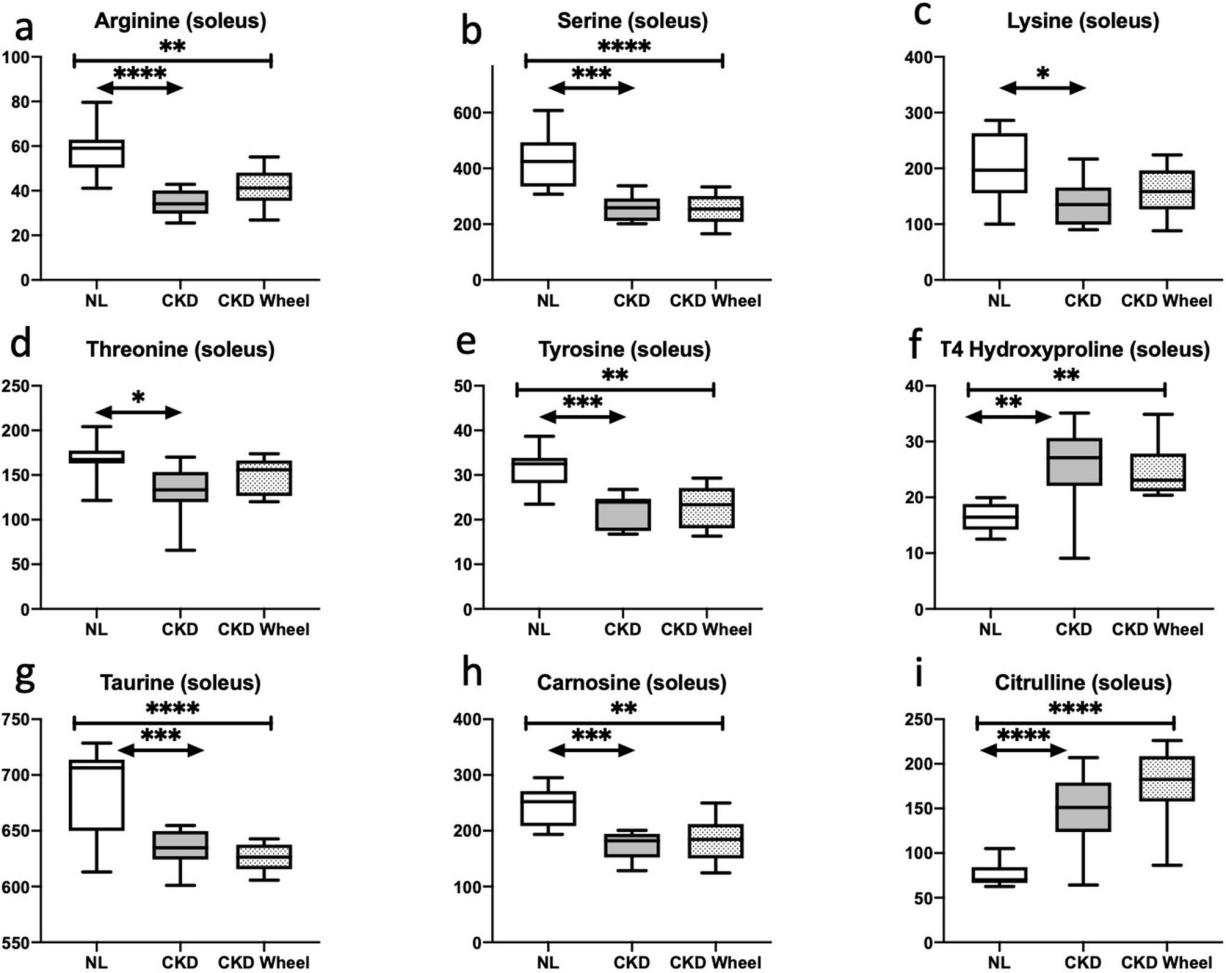
The soleus demonstrated differences in nine amino acid-related compounds. (**a**–**e**) The five standard amino acids (i.e. arginine, serine, lysine, threonine, tyrosine) were reduced. (**f**) The modified amino acid, 4-hydroxyproline was increased in the CKD and CKD-W as compared to NL rats. (**g**–**i**) Taurine and carnosine were reduced while and citrulline was increased in CKD and CKD-W as compared to NL. Data are shown as mean ± SD (n = 9–12 rats each group). *p < 0.05, **p < 0.01, ***p < 0.001. ⎴ (NL vs. CKD comparison); ←  → (NL vs. CKD-W comparison); l—l (CKD vs. CKD-W comparison).

A set of nine amino acid-related compounds were altered in the soleus as shown in Fig. 2. The five standard amino acids (i.e. arginine, serine, lysine, threonine, tyrosine) were reduced (see Fig. 2a–e), while the modified amino acid, 4-hydroxyproline was increased in the CKD and CKD-W as compared to NL rats (see Fig. 2f). This latter modified amino acid plays a role in collagen stability^35^. Taurine and carnosine were reduced, while citrulline was increased in CKD and CKD-W as compared to NL (see Fig. 2g–i). Citrulline is involved in the nitric oxide synthesis pathway which can also be involved in oxidative stress^36^. The increase in citrulline is also consistent with the reduction of arginine as the conversion of arginine to citrulline is catalyzed by nitric oxide synthase. These three amino acid-related compounds may be related to antioxidant activity and oxidative stress, consistent with the anticipated oxidative stress in CKD patients^37,38^, and CKD animals^39^.

**Figure 3:**
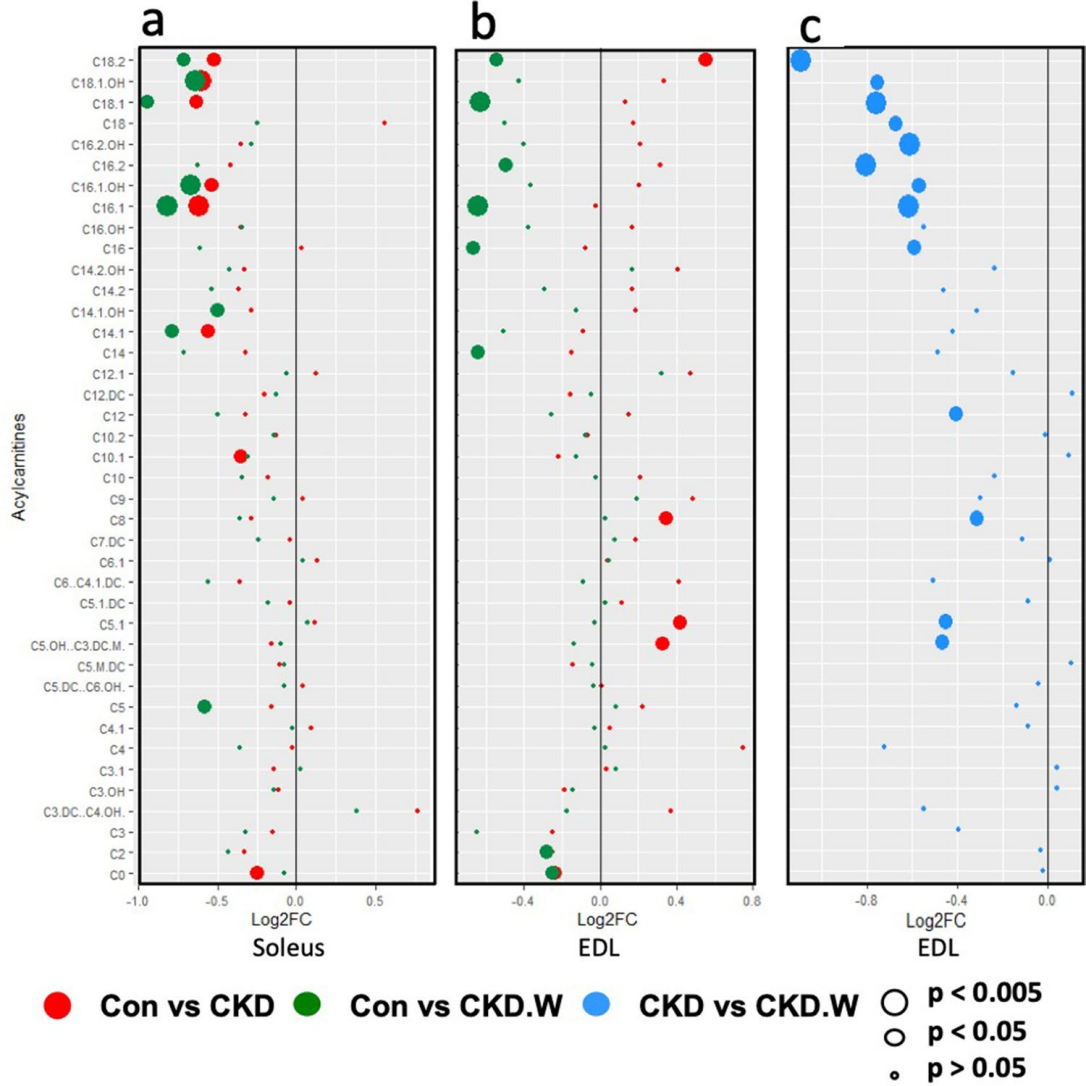
Forest plot of the tissue acylcarnitine levels in the soleus and EDL. The log2 fold changes for the acylcarnitines are plotted for the (**a**) soleus and (**b**,**c**) EDL. The red dots compare the fold change between the NL and CKD rats, green dots between the NL and CKD-W and the blue dots between the CKD and CKD-W. The dot size correlates with the *p* value with the largest dots having *p* values < 0.005, medium dots < 0.05 and small dots > 0.05. The fold comparisons demonstrate group differences where left of center CKD is lower than NL (red dot), CKD-W is lower than NL (green dot), and CKD-W is lower than CKD (blue dot).

The acylcarnitines (AC) are an important class of compounds that provide a surrogate measurement of the functioning of the fatty acid β-oxidation pathway. Figure 3a shows a forest plot of the 40 acylcarnitine species that were profiled. In the soleus, a set of 10 ACs were significantly altered with eight ACs reduced in the CKD group and eight reduced in the CKD-W as compared to NL; six of these metabolites were consistently reduced in both groups. In the forest plot in Fig. 3a most of the AC changes are clustered at the top indicating more pronounced changes in the long-chain ACs.

### Metabolic signatures of CKD and CKD-W rat EDL

**Figure 4:**
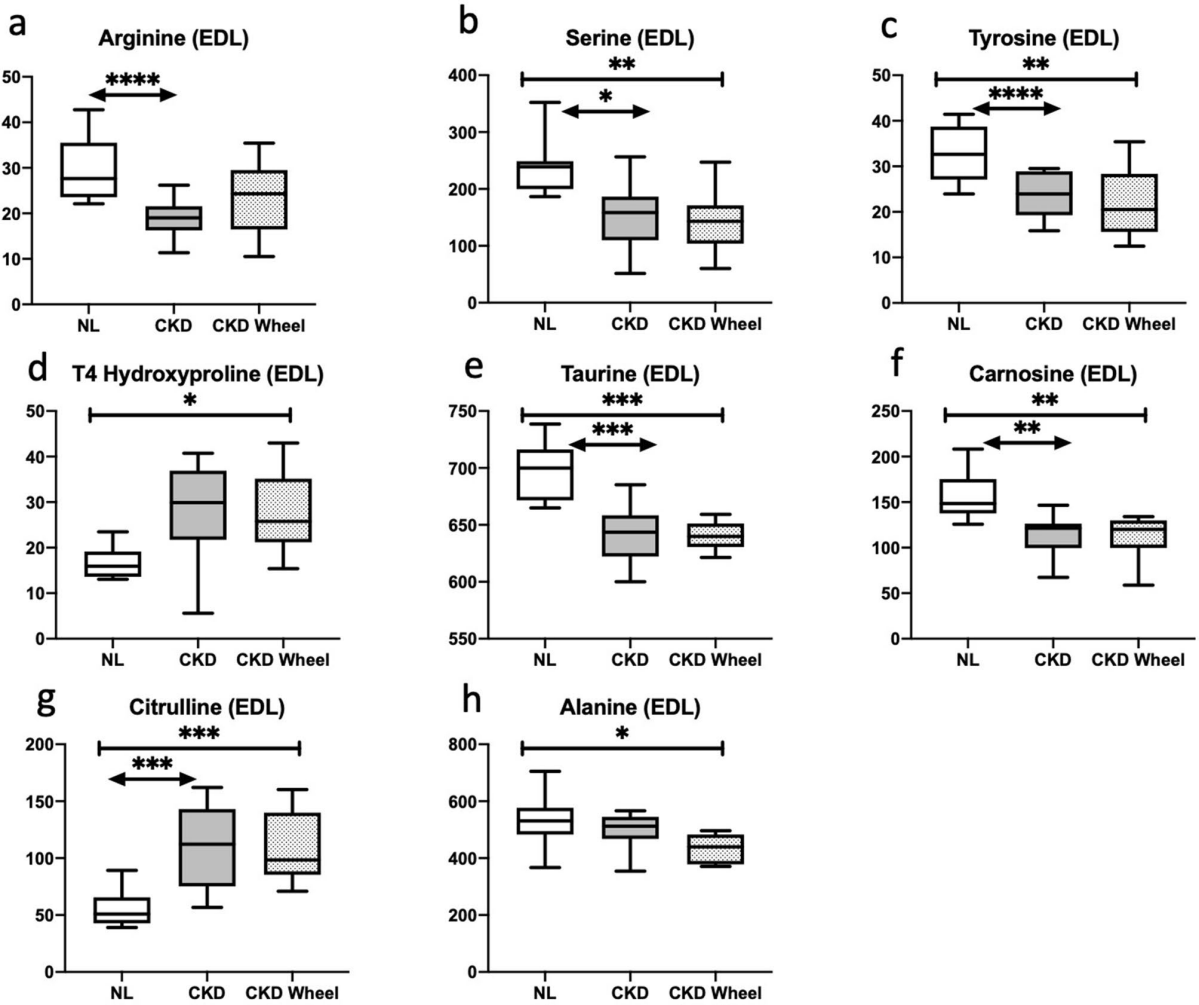
The EDL demonstrated differences in eight amino acid-related compounds. (**a**–**c**) In the EDL, arginine, serine and tyrosine were reduced in both the CKD and CKD-W with the exception of an arginine reduction in the CKD-W group (*p* = 0.09). (**d**) 4-hydroxyproline was increased in the CKD-W group only, no difference between CKD and NL (*p* = 0.056). (**e**–**g**) The potential markers of oxidative stress found in the soleus, including taurine, carnosine and citrulline have the same significant pattern in the EDL. (h) Alanine was reduced in the CKD-W rats compared to NL. Data are shown as mean ± SD (n = 9–12 rats each group). *P < 0.05, **p < 0.01, ***p < 0.001. ⎴ (NL vs. CKD comparison); ←  → (NL vs. CKD-W comparison); l—l (CKD vs. CKD-W comparison).

Analysis of the EDL tissue yielded 67 metabolites which were significantly altered. The heatmap of these changes are shown in Fig. 1b. Both the CKD and CKD-W groups showed 39 significant metabolite alterations with 19 of those changes being common. Unlike the soleus, the EDL had a substantial number of metabolic alterations between the CKD and CKD-W groups with 20 metabolites showing a significant change. Figure 4 shows the alterations in 8 amino acid related species. As in the soleus, arginine, serine and tyrosine were reduced in both the CKD and CKD-W with the exception that in the EDL, the reduction in arginine in the CKD-W group (as compared to NL) was not significant (*p* = 0.09 (see Fig. 4a-c)). Consistent with the soleus, an increase in 4-hydroxyproline is observed in the EDL in the CKD-W group and for the CKD group (*p* value is 0.056, see Fig. 4d). The potential markers of oxidative stress found in the soleus, including taurine, carnosine and citrulline have the same significant pattern as found in the soleus (see Fig. 4e–g). Further, alanine, which is a precursor of carnosine, was reduced in the CKD-W rats compared to NL (see Fig. 4h)^40^.

The major difference in the metabolic profile of the EDL is apparent in the ACs. A total of 16 ACs were different between the groups with five differences in the CKD group, eight in the CKD-W and 13 between CKD and CKD-W. The forest plot in Fig. 3b shows the fold changes for the CKD and CKD-W compared with NL. Carnitine levels are lower in CKD and CKD-W as compared to NL. However, the CKD group demonstrated four increased ACs, while CKD-W demonstrated four decreased ACs as compared to NL. In contrast with the soleus where there were no changes in the ACs between the CKD and CKD-W, the EDL demonstrates significant reductions in 13 ACs (see Fig. 3c). The changes are mainly present in the long-chain ACs with nine of the ACs having chain-lengths of 16–18. This pattern of decreased long-chain ACs with the activity wheel is unique to the EDL.

The overall patterns of metabolic dysregulation suggest significant alterations in amino acid metabolism including effects related to oxidative stress and inflammation in CKD. The significant alterations in the ACs with CKD and the reduction of some of the AC levels in the EDL of the CKD-W group further suggest that derangements in oxidative metabolism in CKD are significantly altered by wheel running. These results led to the exploration of mitochondrial metabolism using tissue respirometry described ahead.

### Reduced skeletal muscle mitochondria in CKD rats following 10-weeks of wheel running

**Figure 5:**
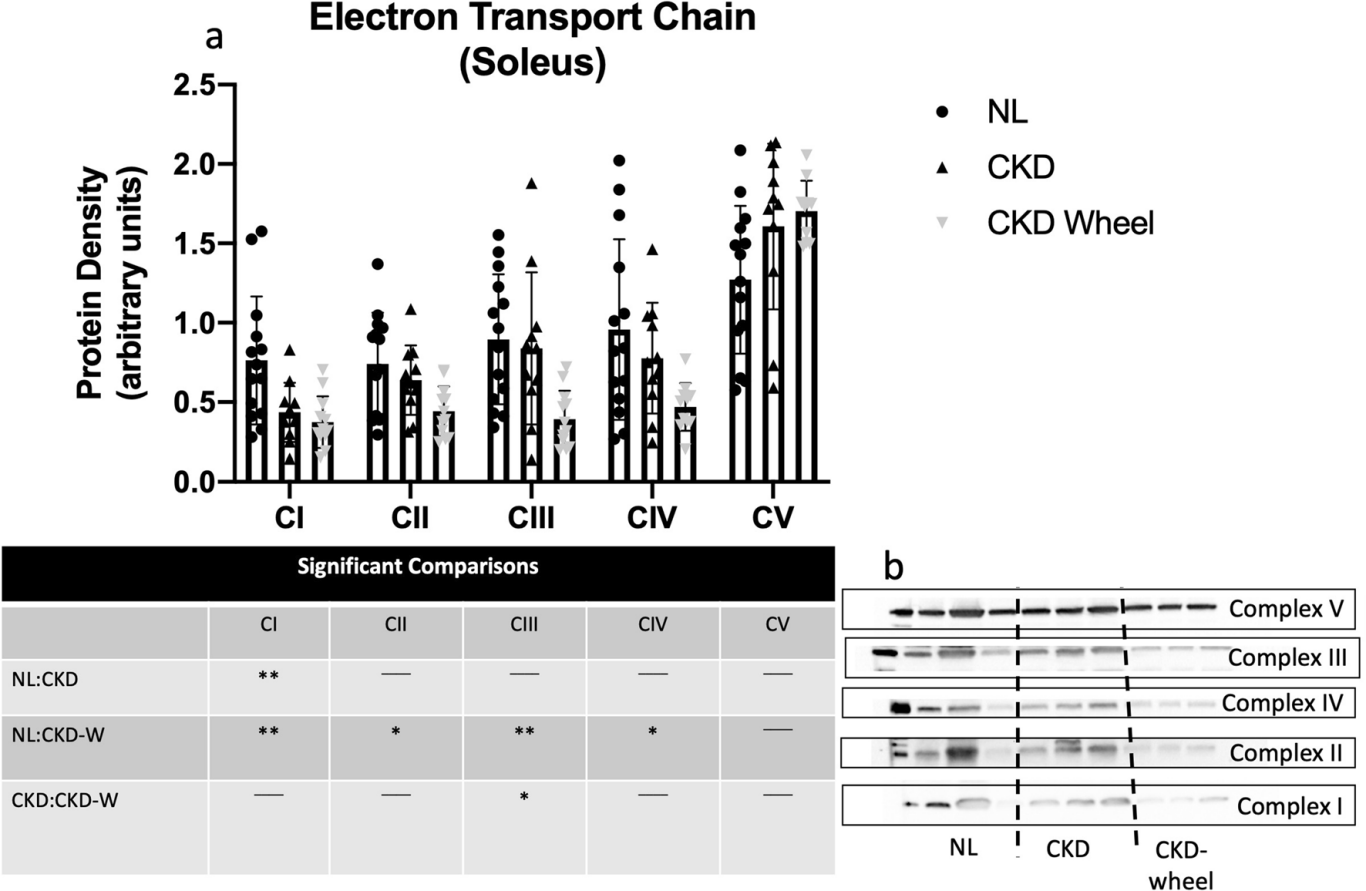
Mitochondrial content is decreased in soleus of CKD wheel running rats. (a) The slow-twitch soleus muscle demonstrated a disease-related reduction in complex I subunit, where CKD was lower than normal; no additional mitochondrial complex markers were different between NL and CKD. However, CKD-wheel rats demonstrated lower mitochondrial content for complexes I-IV subunits when compared to NL rats; only the complex III subunit demonstrated a CKD vs CKD-W difference. (b) Representative OXPHOS blots are provided with manipulated figures to unique exposures times per individual complex. Data are shown as mean ± SD (n = 9 rats each group). *P < 0.05, **P < 0.01.

**Figure 6:**
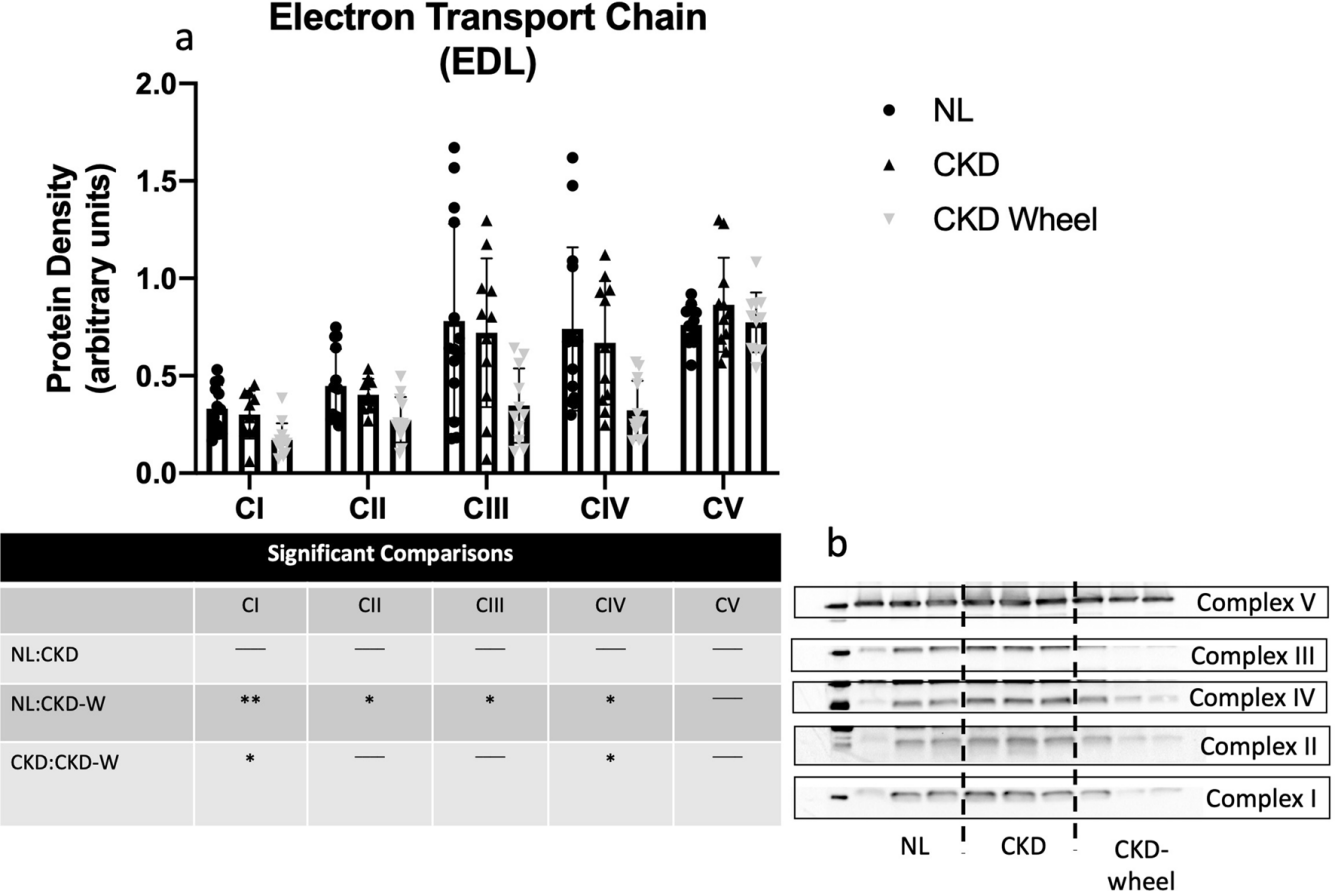
Mitochondrial content is decreased in the EDL of CKD wheel running rats. (**a**) The fast-twitch EDL demonstrated no differences in mitochondrial content markers between NL and CKD rats across complexes I-V subunits. However, when CKD rats performed wheel running, mitochondrial content markers were significantly lower for complex I-IV subunits when compared to NL rats. When comparing CKD to CKD-W, mitochondrial complexes I and IV subunits were lower in CKD-W. (**b**) Representative OXPHOS blots are provided with manipulated figures to unique exposures times per individual complex. Data are shown as mean ± SD (n = 9 rats each group). *p < 0.05, **p < 0.01.

There were reduced markers of mitochondrial content by Western blot in both slow (soleus; see Fig. 5) and fast (EDL; see Fig. 6) twitch muscle fibers in response to 10-weeks of wheel running. The soleus muscle in CKD animals demonstrated a reduction in complex I compared to NL animals, but no differences were observed in complexes II-V. Consistent with EDL, protein content of complexes I-IV were lower in CKD rats compared to NL rats. Only complex III demonstrated a CKD vs CKD-wheel difference. In the EDL muscle, mitochondrial content, as measured by electron transport chain content, was not different between NL and CKD rats, but the levels of complexes I, II, III and IV were significantly reduced in CKD-W compared with NL. Only the changes in complexes I and IV were different between CKD and CKD-W. Mitochondrial porins (aka VDAC) demonstrated a CKD-related reduction in both muscle types and was further lowered by wheel running in the EDL (see Supplementary Fig. S1). These data supported our focus on mitochondrial metabolism and oxidative stress which we have previously shown exists in our model^39^.

### Mitochondrial respiration in skeletal muscle was altered in CKD rats following 10-weeks of wheel running

**Figure 7:**
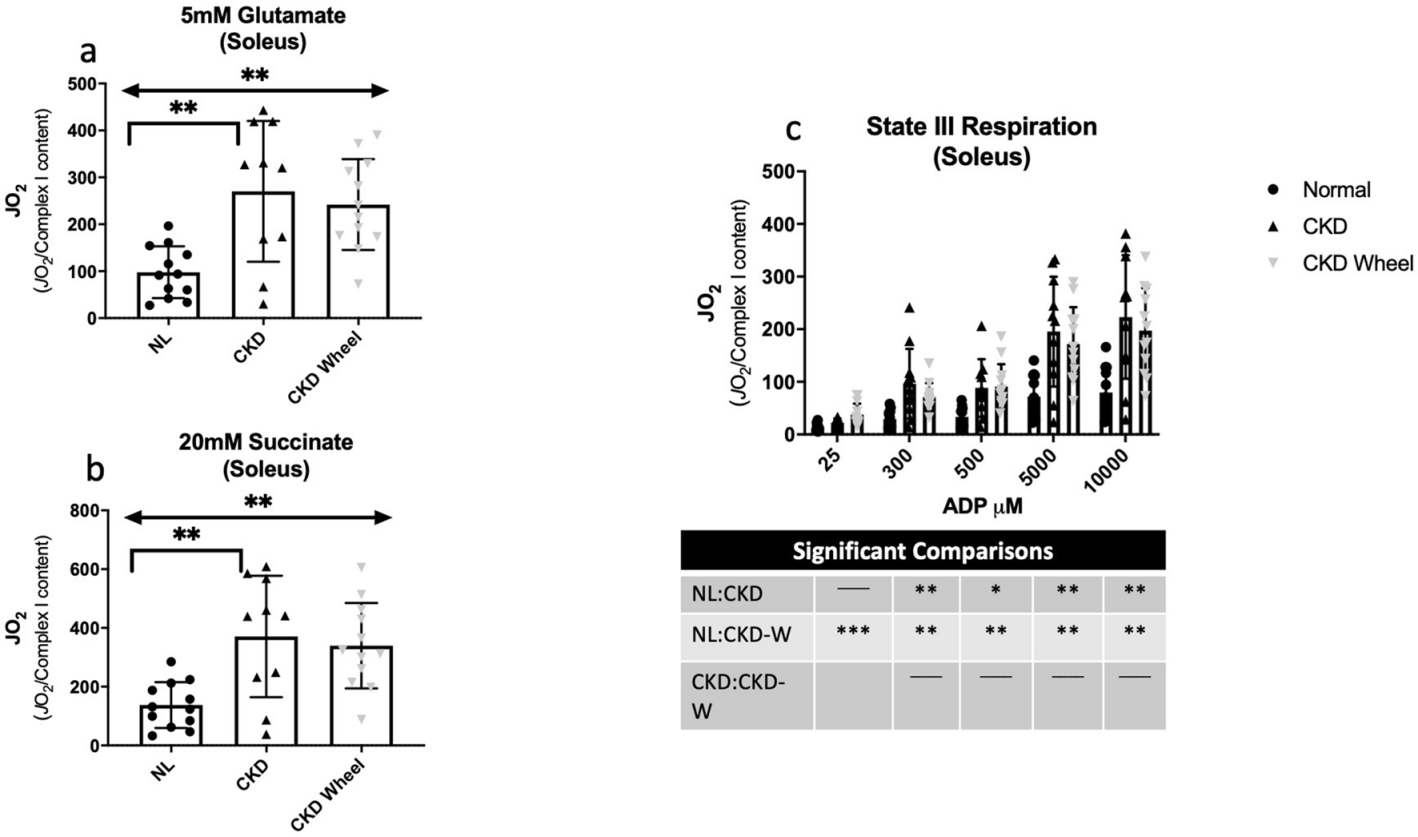
Soleus mitochondrial respiration demonstrated increased respiration in CKD that was not altered by wheel running. (**a**) Maximal ADP-stimulated respiration was increased for CKD soleus as compared to NL, but no difference between CKD and CKD-W with the addition of 5 mM glutamate. (**b**) Complex I + II-supported respiration was similarly increased in CKD as compared to NL, and was not impacted by wheel running following the addition of 20 mM succinate. (**c**) ADP-stimulated respiration (state III) in the soleus was increased in CKD as compared to NL, but was not altered by wheel running**.** Data are shown as mean ± SD (n = 9–12 rats each group). *p < 0.05, **p < 0.01, ***p < 0.001. ⎴ (NL vs. CKD comparison); ←  → (NL vs. CKD-W comparison); l—l (CKD vs. CKD-W comparison).

**Figure 8:**
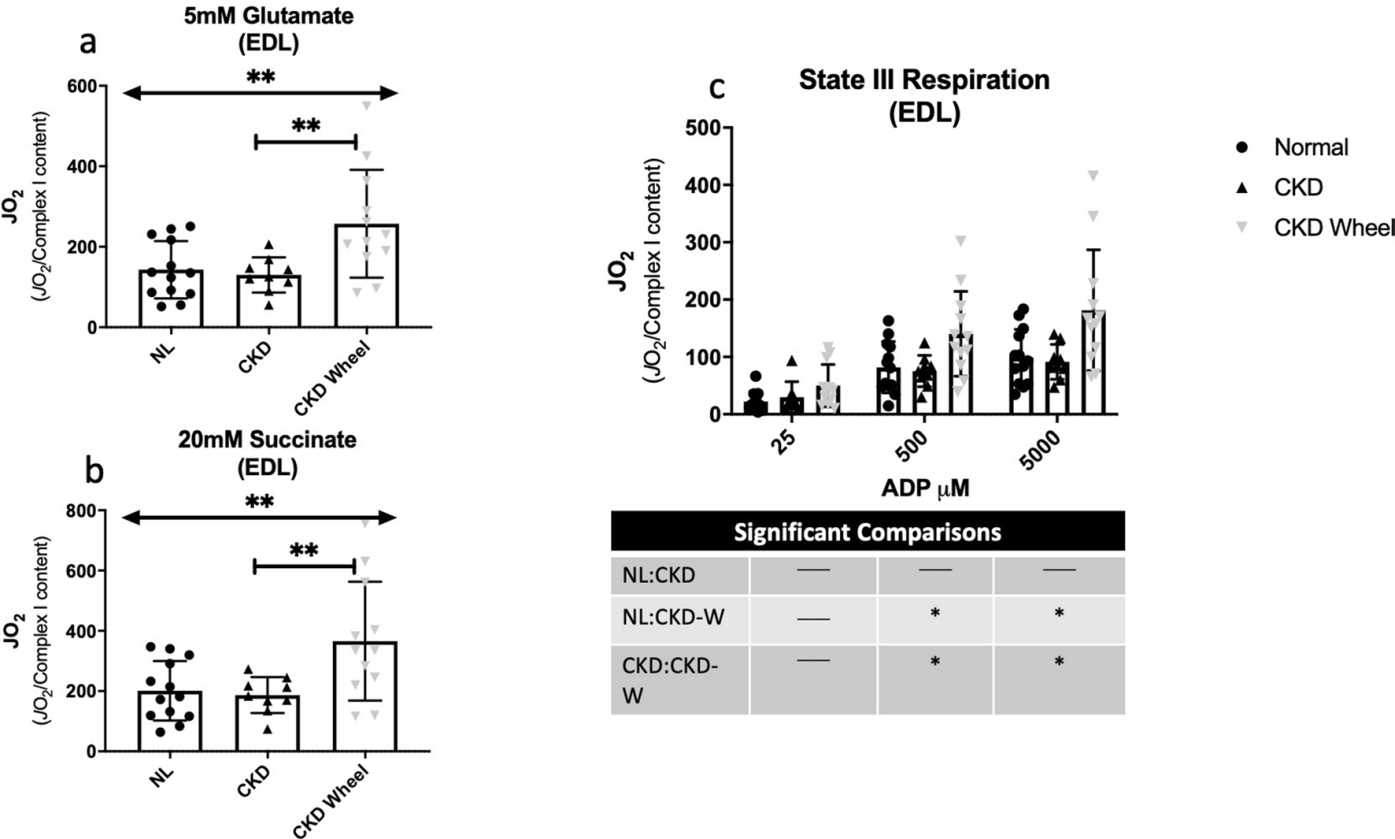
EDL mitochondrial respiration when normalized by Complex I content demonstrated fiber-type specific responses. (**a**) Maximal ADP-stimulated respiration was increased for CKD-W EDL as compared to NL and CKD with the addition of 5 mM glutamate. (**b**) Complex I + II-supported respiration was similarly increased in CKD-W as compared to NL and CKD with the addition of 20 mM succinate. (**c**) ADP-stimulated respiration (state III) in the EDL was increased in CKD-W, but was not altered by CKD alone. Data are shown as mean ± SD (n = 9–12 rats each group). *p < 0.05, **p < 0.01. ⎴ (NL vs. CKD comparison); ←  → (NL vs. CKD-W comparison); l—l (CKD vs. CKD-W comparison).

Tissue respiration measurements normalized by tissue weight are presented for the soleus in Supplementary Figure S2 and for the EDL in Supplementary Figure S3. The soleus muscle in CKD demonstrated greater respiration with ADP concentrations of 300 and 500 μM. The results for the EDL showed no significant respiration differences in response to ADP titrations between the three groups. Given the reduction in complex I content in CKD rats, and the impact of complex I on mitochondrial respiration and downstream complexes (II-V), the data were also analyzed with normalization by complex I content to determine whether specific mitochondrial respiration was altered. Figures 7 and 8 depict the additions of different mitochondrial energy substrates in the soleus and EDL, respectively. In the soleus, glutamate (provokes activity of complex I) and succinate (provokes activity of complex II) demonstrated increased respiration in both CKD and CKD-W as compared to NL rats (see Fig. 7a, b). ADP activated complex I-dependent respiration was increased for CKD and CKD-W compared to NL, with only one exception of no difference between NL-CKD at the lowest ADP concentration (i.e. 25 μM) (Fig. 7c). The EDL demonstrated a similar increase in respiration with the addition of glutamate and succinate (complex I and II respiration; see Fig. 8a,b), but only when comparing CKD-W to either CKD or NL. ADP activated complex I-dependent respiration, at the higher concentrations where more complete saturation of complex I would be expected, demonstrated a significant increase in respiration CKD-W rats as shown in Fig. 8c.

The tissue respirometry data showed some interesting consistencies with the metabolic profiles. In the soleus, the presence of CKD in the rats was responsible for the main metabolic perturbations to metabolism while there were few significant metabolic changes with physical activity. Consistently, the respirometry data found significant differences between the NL and both the CKD and CKD-W rats and no significant impact of physical activity. In contrast, the metabolic profiles of the EDL revealed a significant number of metabolites that were different between the CKD and CKD-W groups, primarily in the acylcarnitine class. An increase in the levels of several ACs were observed in the CKD group while a number were decreased in the CKD-W group, suggesting a change in fatty acid oxidation. The respirometry data demonstrated an increase in oxidative metabolism in the EDL of the CKD-W group, supporting a change toward oxidative metabolism in the CKD-W group.

### Fatty acid/lipid assays

No statistical significance was demonstrated for serum fatty acid quantification (*p* = 0.40) and qualitatively oil red O sections were equivocal with quantifications not pursued further.

## Discussion

The focus of this study was to determine the potential biochemical mechanisms by which voluntary wheel running conferred beneficial effects observed in our previous study CKD-W as compared to CKD: (decreased creatinine 17%, phosphorous 16%, and parathyroid hormone 35%), increased time-to-fatigue 38% (during VO_2max_ testing), reduced cortical porosity and improved bone microarchitecture as previously reported^7^. Despite largely positive effects in our previous study, we found unique effects of wheel running in fast and slow-twitch muscle types. The mostly fast-twitch EDL demonstrated no differences between CKD and NL animals for respiration and mitochondrial complex content. Differences were only demonstrated following 10-weeks of running wheel between CKD and CKD-W, where complex IV content was reduced and respiration increased following glutamate, succinate and ADP additions. In the soleus, CKD as compared to NL demonstrated reduced complex I content and increased following glutamate, succinate and ADP additions. CKD-W demonstrated reduced complex III content and did not impact tissue respiration. The lack of a beneficial effect upon mitochondrial content could speak to an insufficient stimulus provided by wheel running. Wheel running is a voluntary form of exercise that was recently shown to not impact markers of mitochondrial content or dynamics in healthy rats, with the authors recommending exercise of greater intensity^41^. Further, a clinical study found that CKD-related reductions in mitochondrial mass were not impacted by 12-weeks of aerobic or combined aerobic and resistance exercise^25^. Although this was an exercise study, not physical activity, the findings highlight the complexity surrounding activity and exercise prescription. An insufficient intensity is plausible as we have previously demonstrated reduced running wheel distance/day with CKD progression^7^. However, we cannot purely attribute our findings solely to a lack intensity because there were consistent reductions in mitochondrial complex content with activity in both muscle types. Since the reductions were primarily found in response to the running wheel it is plausible that this stimulus resulted in reduced mitochondrial content. In CKD, increasing intensity is therefore complex if low intensity is reducing mitochondrial complex content, and we have previously shown increased muscle catabolism with 12 weeks of progressive treadmill running^28^. We hypothesized that increased oxidative stress was the underlying cause of a lack of exercise-adaptions to treadmill running in a CKD model and could be implicated in the running wheel.

Several recent metabolomics studies of chronic kidney disease have shown alterations in the levels of amino acid and related compounds^42–47^. The kidney directly modulates the levels of serum amino acids through synthesis, degradation, filtration, reabsorption and urinary excretion^48^. The kidney plays a major role in the disposal of glutamine and proline and in the net release of serine, tyrosine and arginine which are synthesized in the kidney for export to other tissues. The reduction in circulating tyrosine in CKD and CKD-W rats may be related to a decrease in the activity of the enzyme, phenylalanine hydroxylase that converts phenylalanine to tyrosine. This enzyme has been observed to be decreased in uremic rats^49^.

A significant increase in citrulline was observed in the serum of both the soleus and EDL of CKD and CKD-W rats. Citrulline can be generated from carbamoyl phosphate in the urea cycle or from arginine in a reaction catalyzed by nitric oxide (NO) synthase^50^. Subsequently, citrulline can be recycled to arginine by the enzymes arginosuccinate synthase (ASS1) and arginosuccinate lyase (ASL). NO has a diverse set of functions in skeletal muscle including satellite cell activation, and regeneration^51^, and overload-induced skeletal muscle hypertrophy^52^. The increased activity of the nitric oxide pathway and the increase in citrulline may also be related to an antioxidant response. Recently, Ham et al. demonstrated that muscle cells treated with citrulline had an increase in mRNA expression of iNOS along with a concomitant increase in antioxidant gene expression including increases in SOD1, SOD3 and catalase^36^. As oxidative stress is involved in the pathogenesis of CKD^53^, this could be a compensatory mechanism. The lack of significant difference in the citrulline levels between the CKD and CKD-W rats indicates that any potential perturbations to the NO signaling pathways in CKD are not impacted by physical activity.

Additional amino acid-related biomarkers of oxidative stress that were affected in the muscle tissue include taurine and carnosine. Taurine is a non-proteogenic that is associated with a diverse range of cellular functions including antioxidant defense mechanisms. A number of studies have reported protective effects of taurine in rodent models of oxidative stress. In diabetic rats, for example, taurine administration led to a significant reduction in the lipid peroxidation marker, malondialdehyde^54–56^. In a study by Dawson et al., the effects of oral taurine administration on exercise-induced oxidative stress was examined in rats. After treadmill running, lipid peroxidation was elevated while taurine supplementation mitigated this effect^57^. In CKD, the depletion of taurine may be the result of the state of oxidative stress which may also contribute to the weaker effects of exercise.

The other metabolic biomarker related to oxidative stress that was depleted in both types of skeletal muscle is carnosine. Carnosine, also known as β-alanyl-L-histidine, is an endogenously synthesized dipeptide engaged in a number of metabolic pathways and has been shown to have reno-protective effects including the reduction of proinflammatory and pro-fibrotic cytokines^58,59^. Carnosine may also function as an antioxidant by scavenging peroxyl and hydroxyl radical^60^. As with taurine, the reduced levels may be the result of utilization to combat the oxidative stress of CKD. Muscle carnosine is synthesized from β-alanine and histidine in a reaction catalyzed by carnosine synthase. Several studies have reported that supplementation with β-alanine can increase exercise performance^61^. As with taurine, the dearth of carnosine may play a mitigating factor in the reduced effects of exercise in CKD.

The acylcarnitines demonstrate the only significant effect of physical activity in the metabolic profiles measured in this study; an effect that was limited to the EDL. Under conditions where mitochondrial stress leads to disturbances in fatty acid oxidation, the result can be a state of incomplete oxidation and an increase in acylcarnitines^62^. In the soleus, a predominantly oxidative slow-twitch muscle, significant decreases in acylcarnitines were observed in both the CKD and CKD-W groups. The main effects were in the long chain ACs suggesting a potential reduction in β-oxidation. Interestingly, in the fast-twitch, glycolytic EDL, the CKD group demonstrated an increase in several medium chain acylcarnitines which would suggest incomplete β-oxidation. In the CKD-W group, there was a shift including significant reductions in carnitine and acylcarnitine (C2) along with a range of longer chain ACs. This suggests that the progression of CKD may reduce the flux through fatty acid oxidation and is not improved with wheel running. Thus, the end result would be a greater reliance upon glycolysis for the EDL.

In this study we do not have direct measures of fatty acid oxidation or glycolysis, but rather indirect evidence of a compensatory mitochondrial response. We found no difference in tissue respiration when normalized by tissue wet weight, but multiple increases in respiration in either CKD or CKD-W; this information is paired with consistent reductions in mitochondrial complex content. The increase in state 3 (ADP-stimulated) respiration when normalized per OXPHOS suggests that the remaining mitochondria, although less in number, retained better function supporting a compensatory response. Specifically, in the soleus compensatory increases were found in ADP-stimulated respiration in CKD that were not altered by wheel running. This is consistent with a study in 5/6 nephrectomy mouse model of CKD, where complex I activity was increased in the gastrocnemius^63^. Although we did not directly assess complex 1 activity, this can be inferred by our results of reduced complex I content paired with increased respiration when normalized by complex I. A compensatory increase in respiration/O_2_ flux was supported by a study that compared skeletal muscle from people with type 2 diabetes, obesity and healthy controls^64^. They found that various mitochondrial proteins were lower in type 2 diabetes and obesity yet mitochondrial respiratory sensitivity to glutamate (NADH, complex I) was greater in type 2 diabetes. While little is known regarding the post-translational mechanisms by which complex I activity can be upregulated, the findings of Larsen et al. and the present study demonstrate that complex I-supported respiration appears to be highly plastic in response to disease stressors. The mechanism of this adaptability requires further investigation.

Physical activity is any movement that requires energy performed by muscle. This umbrella term can include exercise and sport. Studies that perform repeated bodily movements, often in the context of exercise, confer increases in mitochondrial biogenesis, but our data demonstrated that wheel running, in general, lowered expression of mitochondrial content in the soleus and EDL^65,66^. Our findings of lowered mitochondrial content in CKD are consistent with other animal models of CKD that showed reduced mitochondrial content and altered mitochondrial complex activity^10,63,67,68^. The pre-clinical data that suggests a reduced ability to respond/adapt to physical activity was supported by a recent clinical exercise study with non-dialysis dependent CKD patients (stage 3b-5). Skeletal muscle mitochondrial mass was assessed in muscle biopsies collected before and after 12-weeks of either aerobic only or aerobic and resistance training exercise interventions^25^. Mitochondrial mass was reduced at baseline in patients with CKD, and was not improved with either exercise intervention. Mitochondrial mass was assessed by porin expression, which also known as voltage-dependent anion-selective channel (VDAC)^69^. Porin/VDAC is the most abundant protein of the mitochondrial outer membrane and is therefore used as a marker for content. To confirm this response, we assessed multiple mitochondrial membrane transporters and found a similar reduction in VDAC, specifically VDAC2, where CKD was lower than NL and exacerbated by wheel running. Taken together, these preclinical and clinical studies signify a primary defect in mitochondria with CKD that may limit the ability to response and adapt to activity and/or exercise.

In conclusion, our results demonstrate that that the soleus may be more susceptible to CKD-induced changes of mitochondrial complex I content and respiration, while in the EDL, these alterations were in response the physiological load induced by mild physical activity. It is plausible that the intensity of the voluntary wheel running may not have been sufficient to provide an adaptive stimulus for slow fibers and overloading the fast fibers. Future studies should focus on therapies that can improve mitochondrial function in both types of muscle to determine if such treatments can improve activity/exercise tolerance in CKD.

## Methods

### Animal model and tissue harvest

We used the Cy/+_IU_ rat model (CKD rat) with the animals from a previously published study^7^. In brief, male rats were fed standard chow until they were 24 weeks old when they were switched from a standard pellet rat chow to a diet of 18% casein-based protein, 0.7% phosphorus, 0.7% calcium, 5% fat (Harlan Teklan TD.04539) until sacrifice. Female rats were excluded because in this rat model, CKD-mineral bone disease develops spontaneously a much faster progression to end stage renal disease in male animals by 30 to 40 weeks of age, whereas female rats do not develop azotemia even as old as 21 months^70^. Wheel running rats had 24-h access to a voluntary activity wheel (Lafayette Instruments, Model 80850S Scurry Rat Activity Wheel) as previously described^7^. All animals were sacrificed at 35 weeks of age (~ 15% normal GFR) via isoflurane, and tissue procurement and blood collection were performed as previously published^7^. In the primary analyses, we found no effect of wheel running on normal animals, which was expected given the low intensity as defined by running distance and running speed per day^7^. Therefore, for the current study we included three groups (n = 12–14/group): 1) normal littermates (NL); 2) CKD, and; 3) CKD wheel running (CKD-W); animals were randomly assigned to each of the three groups, with female sex the only exclusion criteria. All procedures were reviewed and approved by the Indiana University School of Medicine Institutional Animal Care and Use Committee and methods guided by the ARRIVE guidelines^71^.

### Sample preparation, data collection and processing for metabolomics

Tissue samples for mass spectrometry based metabolomics were prepared per vendor protocols (Biocrates Preparation of Tissue and Fecal Samples for Metabolic Phenotyping, version 1.0). The Biocrates AbsoluteIDQ p180 assay quantifies 187 metabolites from five chemical classes: acylcarnitines, amino acids, biogenic amines, hexoses (sum of hexoses), PCs, and sphingomyelins (SMs). Data were collected on an AB Sciex 4000 QTRAP coupled to an Acquity UPLC system with the selective mass-spectrometric detection using multiple reaction monitoring (MRM) pairs. The amino acids and biogenic amines were detected using and LC-MS/MS method and the lipid species were detected using a flow injection analysis (FIA) MS/MS method per vendor defined settings.

### Mass spectrometry data analysis

Data analysis, including normalization for quantification of metabolite concentrations and quality assessment, were performed with the MetIDQ software package, which is an integral part of the AbsoluteIDQ kit. The metabolite concentration of each metabolite in the series was compared with the measurement detection limit specifications as reported by the manufacturer of the AbsoluteIDQ p180 kit (Biocrates). A metabolite was excluded from further analyses if its concentration measurement data did not meet all of the following criteria: (1) minor of 20% of missing values (non-detectable peak) for each quantified metabolite in each experimental group (2) 50% of all measured sample concentrations for the metabolite had to be above the limit of detection (LOD). Animals grouping were provided following sample analysis.

### Mitochondrial respiration

The EDL and soleus were prepared for respiration with modifications as previously published^72^. In brief, high‐resolution O_2_ consumption measurements were performed in 2 ml of respiration medium (Buffer Z solution) using the Oroboros Oxygraph‐2 k (Oroboros Instruments, Corp., Innsbruck, Austria). 5 mM pyruvate and 5 mM malate were added as complex I substrates (via generation of NADH to saturate electron entry into complex I) to initially stimulate respiration in the absence of ADP (State II) which is a measure of proton leak, and an index of uncoupled respiration. This was followed by ADP titrations in stepwise increments (25 µM-10 mM ADP, State III respiration), followed by 5 mM Cytochrome *c*, 5 mM glutamate (NADH to further saturate Complex I) and 20 mM succinate (FADH_2_ to support Complex II). Respiration data was normalized by two methods: 1- wet bundle weight only, 2- wet bundle weight divided by Complex I NDUFB8 subunit per western blot data to provide JO_2_/Complex I content respiration. The complex I normalization approach permits comparisons between respiration per mg of muscle which may be influenced by changes in mitochondrial content vs intrinsic respiratory changes within mitochondria themselves independent of content changes.

### Western blot

Western blotting was performed as previously described^39^ using the EDL and soleus lysates stored at − 20 °C. Protein expression was assessed via commercially available monoclonal antibodies for the following: electron transport chain protein (rodent OXPHOS cocktail, Abcam, ab110413, Cambridge, UK, 1:500 dilution) and VDAC 2 (voltage-dependent anion channel, Cell Signaling, #9412, Danvers, MA, 1:500 dilution). The primary antibody was maintained at 4 °C overnight followed by incubating with peroxidase conjugated secondary antibody (1:5000 dilution), and immunodetection with the Enhanced Chemiluminescence Prime Western Blot Detection Reagent (Amersham, Piscataway, NJ). The band intensity was analyzed by ChemiDoc MP Imaging System (Imaging Lab 4.0, Bio-Rad, Richmond, CA) and normalized to total protein expression using Ponceau S (Santa Cruz Biotechnology, Santa Cruz, CA).

### Free fatty acid assay

For secreted FFA measurement, 50uL of serum was collected and measured by FFA assay kit (Sigma-Aldrich, MAK044-1KT) following the instructions from the company.

### Oil red O staining

Cryostat cut transverse cross-Sects. (10 μm) were characterized by Oil Red O as previously performed^73^, which stains the lipid droplets in the cytoplasm^74^.

### Statistics

Our a priori question was aimed at whether voluntary wheel running can alter or normalize rats with CKD. Therefore, we included three groups in this study, NL, CKD, and CKD-wheel using a one-way ANOVA with a Tukey post-hoc analysis for data that passed the normality test (GraphPad Prism 8, GraphPad Inc.). If the normality assumption failed via the D'Agostino & Pearson test, data was analyzed via a Kruskal–Wallis test with Dunn's multiple comparisons test for post-hoc analysis. The metabolomics data were analyzed in the open-source software package Viime (viime.org) with one-way ANOVA with Tukey post-hoc testing.

## Supplementary Information

Supplementary Information
