# Supplementary material for: Skeletal muscle metabolic responses to physical activity are muscle type specific in a rat model of chronic kidney disease
Source: Sci Rep. 2021 May 7;11:9788. doi: 10.1038/s41598-021-89120-8 (PMC8105324; doi:10.1038/s41598-021-89120-8)
Supplement: Supplementary file 1 — Supplementary Information [file 41598_2021_89120_MOESM1_ESM.pdf]

Title: Skeletal muscle metabolic responses to exercise are fiber type specific in a rat model of Chronic Kidney Disease

Authors: **Avin KG, Hughes MC, Chen NX, Srinivasan S, O'Neill KD, Evan AP, Bacallao RL, Schulte ML, Moorthi RN, Gisch DL, Perry CGR, Moe SM, O'Connell, TM

Addresses and Affiliations

[1,2,3]Keith G. Avin (keigavin@iu.edu)

[4]Meghan C. Hughes (hughesmeghanc@gmail.com)

[1,3]Neal X. Chen (xuechen@iu.edu)

[1,3]Shruthi Srinivasan (shrusrin@iu.edu)

[1,3]Kalisha D. O'Neill (kaddingt@iu.edu)

[5]Andrew P. Evan (aevan@iupui.edu)

[1,3]Robert L. Bacallao (rbacalla@iu.edu)

[6]Mike L. Schulte (schultml@iu.edu)

[1] Ranjani N. Moorthi (rmoorthi@iu.edu)

[7]Debora L. Gisch (debora.gisch@gmail.com)

[4]Christopher G.R. Perry (cperry@yorku.ca)

[1,3,5]Sharon M. Moe (smoe@iu.edu)

[8] Thomas M. O'Connell (thoconne@iu.edu)

[1]Division of Nephrology, Indiana University School of Medicine, Indianapolis, IN, United States.

[2]Department of Physical Therapy, Indiana University School of Health and Human Sciences, Indianapolis, IN, United States.

[3]Roudebush Veterans Affairs Medical Center, Indianapolis, IN, United States.

[4]School of Kinesiology and Health Science, Muscle Health Research Centre, York University, Toronto, ON, Canada

[5]Department of Anatomy and Cell Biology, Indiana University School of Medicine, Indianapolis, IN, United States.

[6]Department of Radiology and Imaging Sciences, Indiana University School of Medicine, Indianapolis, IN, United States.

do Rio Grande do Sul, Departamento de Engenharia Mecânica, Porto Alegre, Brasil

[8]Department of Otolaryngology, Head & Neck Surgery, Indiana University School of Medicine, Indianapolis, IN, United States.

Corresponding Author

Keith G. Avin

keigavin@iu.edu

Division of Nephrology

Indiana University School of Medicine

950 W. Walnut St.

R2 202

Indianapolis, IN 46202

Figure S1

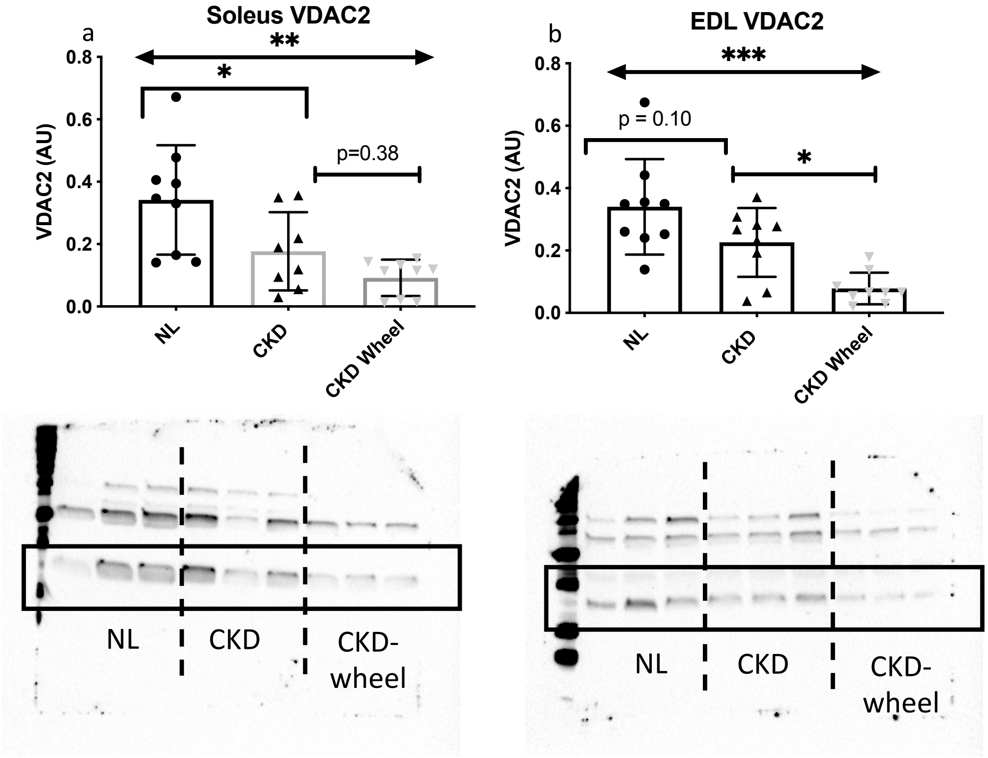
a
Soleus VDAC2
**
*
p=0.38
VDAC2 (AU)
0.8
0.6
0.4
0.2
0.0
NL
CKD
CKD Wheel
b
EDL VDAC2
***
p = 0.10
*
VDAC2 (AU)
0.8
0.6
0.4
0.2
0.0
NL
CKD
CKD Wheel
NL
CKD
CKD-wheel
NL
CKD
CKD-wheel

Figure S2

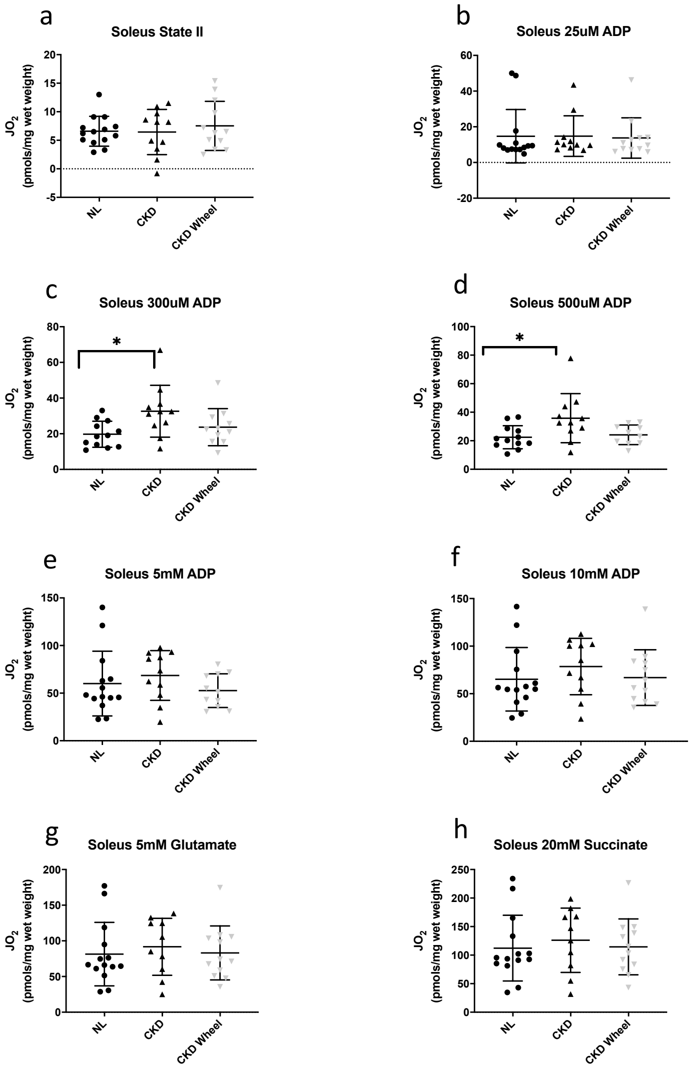
a
Soleus State II
JO2 (pmols/mg wet weight)
NL
CKD
CKD Wheel
b
Soleus 25uM ADP
c
Soleus 300uM ADP
*
d
Soleus 500uM ADP
*
e
Soleus 5mM ADP
f
Soleus 10mM ADP
g
Soleus 5mM Glutamate
h
Soleus 20mM Succinate

Figure S3

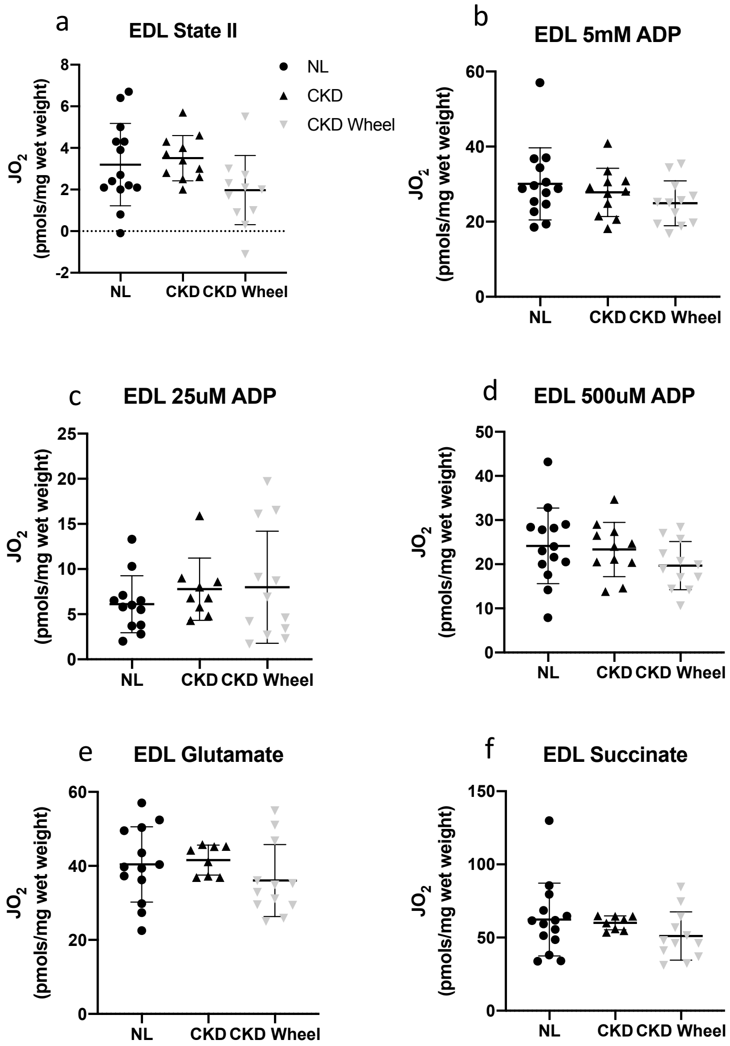
a
EDL State II
NL
CKD
CKD Wheel
JO2 (pmols/mg wet weight)
8
6
4
2
0
-2
NL
CKD
CKD Wheel
b
EDL 5mM ADP
60
40
20
0
c
EDL 25uM ADP
25
20
15
10
5
0
d
EDL 500uM ADP
50
40
30
20
10
0
e
EDL Glutamate
60
40
20
0
f
EDL Succinate
150
100
50
0

Figure S4

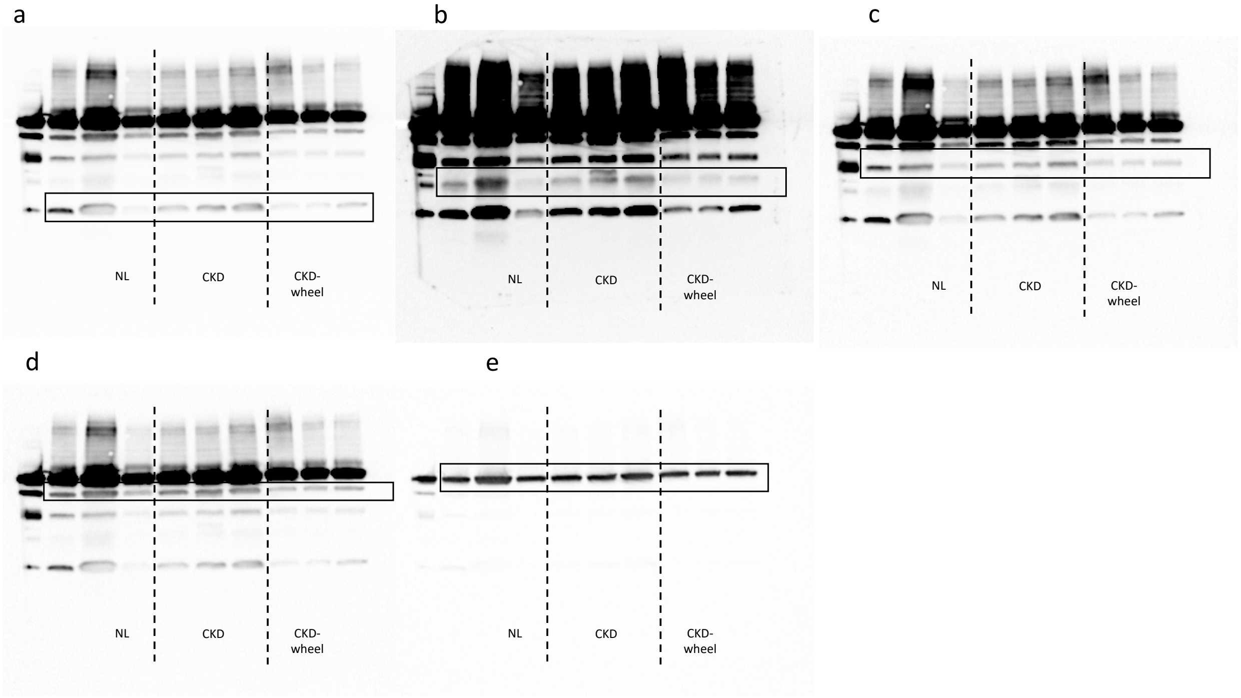

Figure S5

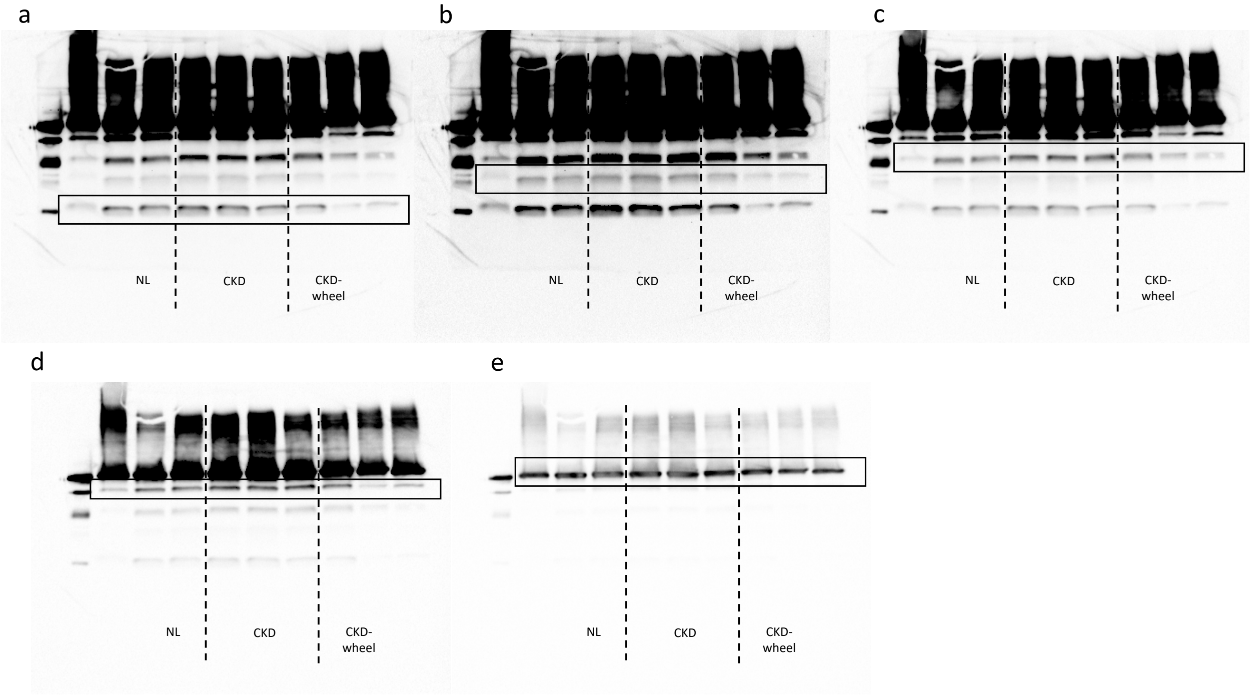

Supplementary Figures Legend

Figure S1. Mitochondrial solute transport marker VDAC2 is reduced following wheel running in CKD rats. (a) In the soleus, VDAC2 expression was significantly lower in the CKD-W rats compared to NL. The disease effect was trending towards significance when comparing CKD to NL. (b) In the EDL, CKD-W rats demonstrated significantly reduced VDAC2 expression when compared to NL or CKD rats. Data are shown as mean ± SD (n=9 rats each group). *P<0.05, **P<0.01, ***P<0.0001. ⌐ (NL vs. CKD comparison); ←→ (NL vs. CKD-W comparison); l—l (CKD vs. CKD-W comparison).

Figure S2: Soleus mitochondrial respiration was greater in CKD at exercising concentrations of ADP. CKD rats demonstrated greater respiration at 300 and 500 μM ADP when

compared to NL. However, there were no significant differences in respiration for the remaining substrates when comparing NL, CKD or CKD-W. Data are shown as mean ± SD (n=12-14 rats each group).

Figure S3: EDL mitochondrial respiration when normalized by tissue weight is not different with CKD. There were no significant differences in respiration in response to the substrates when comparing NL, CKD or CKD-W. Data are shown as mean ± SD (n=12-14 rats each group).

Figure S4: Representative Original Soleus OXPHOS Blots: Original unmanipulated figure of Soleus (a) 20kDa Complex I, (b) 30kDa Complex II, (c) 40kDa Complex IV, (d) 48kDa Complex III, (e) 55kDa Complex V.

Figure S5: Representative Original EDL OXPHOS Blots: Original unmanipulated figure of EDL (a) 20kDa Complex I, (b) 30kDa Complex II, (c) 40kDa Complex IV, (d) 48kDa Complex III, (e) 55kDa Complex V.
